# 

**Published:** 1873-01

**Authors:** 


### TABLE OF CONTENTS.

ORIGINAL COMMUNICATIONS.

What May be Really Learned by Microscopic Examinations of Urine. By James Tyson, M.D., . . . . . . . . . . . . . 1
Diphtheria. By W. T. Taylor, M.D., . . . . . . . . . . 5
Correspondence. By T. J. Heard, M.D., . . . . . . . . . 10

SELECTIONS.

Chlorine Water in the Treatment of Diphtheria. By W. G. Balfour, M.D., 12
The Action of Belladonna Upon the Heart and Arteries, and Upon the Heart in Pericarditis and Endocarditis. By David Wooster, M.D., . . . 14
Three Cases of Rachitis, . . . . . . . . . . . . . 16
Dyspepsia of Liquids. By Dr. John C. Thorowgood, . . . . . . 19
Conjunctivitis Granulosa Chronica Treated by Galvanization. By C. W. Trueheart, M.D., . . . . . . . . . . . . . 21
Acute Myringitis—Atropine in its Treatment. By A. D. Williams, M.D., . 23
Injurious Effect of Carbolic Acid from Injudicious Local Application. By John S. Hughson, M.D., . . . . . . . . . . . . 26
A New Mode of Treating Puerperal Fever. By Dr. Charles Bell, . . . 28
On Lienteric Diarrhœa in Children. By Eustace Smith, M.D., . . . 31
Treatment of Erysipelas, . . . . . . . . . . . . . 34

EXTRACTS AND GLEANINGS.

Fever, Treatment of, 35; M. Ollier's Occlusive Dressing, 37; Ice to the Neck in Hysteria, and in Suspended Respiration, 38; A Simple Method of Treating Chronic Diarrhœa—For Chilblains, 39; Two Cases of Malarial Diarrhœa—Glycerin Lotion, 40; Syphilitic Ulceration of the Face—Coffee as a Vehicle for Quinine, 41; Treatment of Scarlet Fever—Subcutaneous Injection of Mercury in Syphilis, 42; The Relation of Obesity to Diseases of the Sexual Organs in Women—Cancrum Oris, 43; Application of Electricity—Night Sweats, 44; A Mode of Operating for Radical Cure of Varicocele—Uterine Hemorrhage—Novel Method of Detecting Small Pox—Castor Oil Emulsion, 45; Galvanic Treatment of Bed-Sores and Indolent Ulcers—Blisters in Pneumonia—Treatment of Gonorrhœa, 46; Rapid Death from Stings of Bees—Changing the Direction of Wild Hairs, 47; Treatment of Constipation by Electro-Therapeutics—Electricity in Angina Pectoris—Abortive Treatment of Boils and Whitlow—Bromide of Calcium, 48; A Dangerous Paper—Aspiration and Iodine Injection in Catarrh of the Lachrymal Sac—Transplanting a Tooth, 49; Aspiration in Hydrocephalus—Hooping-Cough—Incontinence of Urine in Children; Arsenic in Red Paper Hangings, 51; To Prevent Abrasion of the Cheek from Spiral Springs—Preserving Vaccine Virus—Fluid to Restore Putrid Tissues—Carious Teeth, 51; Herpes Zester Treated by Electricity—Cancer—Carbolic Acid in Gonorrhœa—Blood in the Urine—Whitlow, Carbolic Acid, 52; Bromide of Iron—Injection of Alcohol into Cysts—Chronic Gastritis—Treatment of Ganglion, 53; Knot in the Cord—Dust in the Air-Passages—Animalculæ in Buttermilk—Syphilis, 54; Cundurango is not Dead yet—Amputation of the Cervix Uteri by Means of the Galvanic Cautery—Carbolic Acid in Pruritis, 55; Effects of Color on Health—Ipecacuanha in Epistaxis, 56; New Method of Making Beef-Tea—Sunstroke, 57; Tincture of Calabar Bean—Antidotes to Carbolic Acid—Constipation, 58.

EDITORIAL AND MISCELLANEOUS.

Our New Name—Our Exchanges, . . . . . . . . . . 59
Communications—Clinical Reports from Private Practice—Our Associate and Corresponding Editors—The Union Oil Stove, . . . . . . . 60
Savannah Medical College, Address of Professor J. G. Thomas—West Virginia Medical Society—A Model Parlor Magazine, . . . . . . 61
The Medical Society of Virginia, . . . . . . . . . . . 62
The Best Boys' and Girls' Magazine, . . . . . . . . . . . 63

